# Modern health worries: Deriving two measurement invariant short scales for cross-cultural research with Ant Colony Optimization

**DOI:** 10.1371/journal.pone.0211819

**Published:** 2019-02-07

**Authors:** Gabriel Olaru, Oliver Wilhelm, Steven Nordin, Michael Witthöft, Ferenc Köteles

**Affiliations:** 1 Psychological Assessment, University of Kassel, Kassel, Germany; 2 Individual Differences and Psychological Assessment, Ulm University, Ulm, Germany; 3 Department of Psychology, Umeå University, Umeå, Sweden; 4 Department of Clinical Psychology, Psychotherapy, and Experimental Psychopathology, Johannes Gutenberg-University, Mainz, Germany; 5 Institute of Health Promotion and Sport Sciences, ELTE Eötvös Loránd University, Budapest, Hungary; Universidad de Tarapaca, CHILE

## Abstract

Worries about possible harmful effects of new technologies (modern health worries) have intensely been investigated in the last decade. However, the comparability of translated self-report measures across countries is often problematic. This study aimed to overcome this problem by developing psychometrically sound brief versions of the widely used 25-item Modern Health Worries Scale (MHWS) suitable for multi-country use. Based on data of overall 5,176 individuals from four European countries (England, Germany, Hungary, Sweden), Ant Colony Optimization was used to identify the indicators that optimize model fit and measurement invariance across countries. Two scales were developed. A short (12-item) version of the MHWS that represents the four-factor structure of the original version and an ultra-short (4-item) scale that only measures the general construct. Both scales show that overall levels of health worries were highest in England and Hungary, but that the main reason for concern (e.g. electromagnetic radiation or food related fears) differs considerably between these countries. This study also shows that even if measurement invariance of translated self-report instruments across countries is problematic, it can be optimized by using adequate item selection procedures. Differences of modern health worries across countries and recommendations for cross-cultural research are discussed.

## Introduction

Worries about potentially adverse health effects of scientific and industrial progress appear to be longstanding phenomenon. Prominent examples are health complaints related to railroad accidents known as the ‘railway spine syndrome’ in the early 19^th^ century as well as the more recent phenomenon of ‘electric allergy’ [1]. With the start of the third millennium, these “modern health worries” (e.g. worries about adverse health effects of genetically modified food) have become a focus of psychometric research in health psychology and behavioural medicine. However, cross-cultural research on modern health worries is lacking. For an unbiased comparison of across countries, measurement invariance needs to be established before mean-level differences can be analysed [2]. This paper summarizes the current state of knowledge concerning modern health worries (MHWs), as assessed by the Modern Health Worries scale (MHWS) [3]. We subsequently present how measurement invariance of the scale can be improved using Ant Colony Optimization [4–6]. The derived short scales are used to investigate country specific differences in MHWs. Recommendations and advantages of this procedure compared to classical approaches for dealing with a lack of measurement invariance (e.g. partial measurement invariance) are subsequently discussed.

### Origins, prevalence, and correlates of MHWs

The construct of MHWs is defined as “the degree to which individuals are concerned about features of modernity affecting their health” [3]. An increase in health awareness as well as media reports focusing on toxic and environmental health threats might be responsible for the reported increase in MHWs in Western countries. Media reports on environmental threats (e.g. low-dose environmental chemicals, weak electromagnetic fields) are typically sensational and one-sided [7]. Their causal role in an increase of MHWs was supported by empirical findings [8].

MHWs are positively associated with facets of psychopathology. In a study with 757 college students, small to medium sized associations were found with depressive symptoms, health related anxiety, and somatization [9]. In a study with young secondary school students (n = 480), small positive associations were observed with trait anxiety, health anxiety, and somatization [10]. Petrie et al. [3] reported a weak association between MHWs and trait negative affect in a sample (n = 526) of college students. However, associations with trait negative affect were not confirmed in later studies [11], showing that negative affect might not be a core feature of MHWs. Lahrach and Furnham [12] found a weak negative correlation with the quality of self-perceived mental health. In the same study, a medium sized association with the strength of medical conspiracy theory beliefs was found, which is in line with previous results of associations between MHWs and tendencies towards a holistic world-view, characterised by spirituality and believing in astrology [13]. In sum, although positive associations exist between MHWs and facets of psychopathology (particularly those that are marked by negative affect, e.g., depression and anxiety), these associations appear smaller than expected by the term “worries”. From a clinical psychological perspective, MHWs seem to be stronger related to a holistic-experiential thinking style, which manifests itself in openness to paranormal beliefs and pseudoscientific theories (which might reflect mild variants of schizotypal traits) compared to internalizing psychopathology [12].

Perhaps most importantly, MHWs are associated with subjective health complaints [14] and non-specific somatic symptoms [15], and predict symptoms in longitudinal studies [11]. Similarly, associations with the utilization of health care services and sick leave have been reported [16].

### Assessment and dimensionality of MHWs

Although other questionnaires have also been developed to assess environmental concerns [17], the most widely used instrument in the field is the MHWS developed by Petrie and colleagues [3]. The first published version of the scale consisted of 25 items and was characterized by a four factor structure. The factors are *Toxic interventions* (11 items), *Environmental pollution* (6 items), *Tainted food* (5 items), and *Radiatio*n (3 items). The MHWS has been translated to German [9], Hungarian [18], and Swedish [19] amongst other translations. The correlated factor structure of the 25-item scale was replicated with both exploratory [9] and confirmatory [19,20] factor analysis (with the inclusion of correlated residuals). Longer versions were inconsistent with respect to number and content of the factors [12,21], which might have partly resulted from the orthogonal rotation applied in these two studies. Brief versions of the MHWS have also been developed measuring the general construct with nine to ten item [22].

The correlated four-factor structure is currently the most widely used model of the MHWS. However, most studies on MHWs typically compute and report a general MHWs score across all four factors. This general factor for the 25-item scale has not been yet evaluated with factor analytic procedures. The high correlations between the four factors reported in previous studies using confirmatory factor analysis (r = .57-.87) [19,20] suggest the presence of a general factor atop of the four domain factors. To provide more insight into the dimensionality of the MHWS, we compare a higher-order model of MHWs to the correlated four-factor model in this study.

In addition to issues on the dimensionality of MHWs, the use of different versions of the scale makes the comparison of results difficult between studies and countries. This is most problematic in regard to the existing short scales, which neglect the more specific factor level. Group differences on the more detailed level will thus be overlooked. Moreover, none of the existing scales was tested for measurement invariance across countries. As country-specific differences in the strength of various worries might exist, a widely usable version with an acceptable level of measurement invariance is needed. The goal of this study is thus to create a short version of the MHWS that retains the factor level and is measurement invariant across countries. Model fit of self-report measures is often problematic due to cross-loadings and residual correlations, and a common procedure to overcome these issues is to estimate the models on the basis of aggregates of the manifest indicators [23], by data-driven freeing of constraints on problematic indicators, or by applying less restrictive modeling techniques, such as Exploratory Structural Equation Modeling (ESEM) [24–26]. Similarly, problems with the measurement invariance of self-report measures may occur due to translation issues or cultural differences [27]. This problem is often addressed by parceling indicators or freeing equality constraints on problematic items (also known as partial invariance). The issue with these approaches is that they only conceal or incorporate problems of the scale into the model rather than eliminate them. While model fit may go up due to lower restrictions in the model after parceling or freeing model constraints, model parameters will still be biased if model fit and measurement invariance of the initial model were inadequate. Data driven modifications to the model are also often theoretically hard to justify and affect the generalizability of the model. While ESEM is a great tool to relieve the stress imposed by the strict cross-loading constraints in CFA, the resulting cross-loadings can be very high and thus require a theoretical justification, or can indicate an inherent flaw of the scale. This is particularly problematic in the case of multi-group models, where these additional cross-loadings also need to be measurement invariant across groups. Good model fit alone does not correspond to good validity. A sound theoretical foundation and adequate model structure is also required for the scale scores to be meaningful representations of the latent constructs. As such, we used an alternative procedure in this study: Instead of modifying the model to fit the scale, we want to select the items that support the theoretical model of MHWs and are most measurement invariant across countries. By eliminating problematic items instead of retaining them, a much less biased comparison of model parameters is possible across groups. One such item selection procedure that is able to simultaneously optimize model fit and measurement invariance—as well as a wide range of other criteria—is Ant Colony Optimization (ACO) [4–6].

### Item selection with Ant Colony Optimization

ACO is a meta-heuristic optimization procedure that finds the optimal (or close-to-optimal) solution similar to the way ants find the shortest route between nest and food source. Ants use pheromones to mark routes to the food source and attract other ants to the route. The shorter the route, the faster pheromones can accumulate. This will in turn attract more ants until all ants follow the shortest route [28]. ACO is an adaptation of this natural phenomenon that uses virtual pheromones to increase the attractiveness of item sets (= route) that yield better psychometric properties (e.g., model fit). After randomly selecting items and comparing the psychometric properties of the selected item sets, ACO will increase the virtual pheromones of the “best” items belonging to the best item sets. This, in turn, increases the likelihood of these items to be selected in subsequent iterations. This process is repeated until a predefined criterion or number of iterations is reached. ACO has proven to be a purposeful selection procedure for optimizing absolute model fit and reliability [29,30], as well as measurement invariance [5,6]. In contrast to classical selection procedures (e.g., selecting items based on high main loadings), ACO is able to optimize several scale-level criteria simultaneously (e.g. model fit and reliability) [31]. Instead of removing items sequentially, as it is typically done in classical test shortening procedures (e.g., remove items based on highest “Cronbach’s alpha if an item is deleted”), ACO searches for item samples of a fixed size. Thus, it is not affected sequence effects and more likely to find the best model instead of optimize the scale towards a local optimum. Metaheuristic approaches, such as ACO, also have the benefit of being computationally much less demanding than examining all possible item combinations of a fixed size.

### Aims of the study

The current study aims to develop two short versions of the MHWS that are measurement invariant across four European countries. First, we compared a higher-order factor of MHWs to the correlated factor model. Second, we developed a version that preserves the four-factor structure of the original scale; and third, we derived an ultra-short version for the assessment of the general construct. In addition, we wanted to explore possible differences in the strength of modern health worries across the four countries.

## Materials and methods

### Participants

Non-student data from previous MHWs studies with a total number of 5,211 participants were used. Studies were conducted in four European countries (England, Germany, Hungary, Sweden) and were approved by the authorized ethical boards. We removed 35 cases due to missing values on at least one item of the MHWS scale. Sample characteristics and origins of the remaining 5,176 cases are presented in Table 1.

**Table 1:** Sample characteristics and origins.

			age		gender	
Country	Sample	*N*	*M*	*SD*	male	female
Hungary	[18]	127	49	18	46	99
	[32]	103	43	18	37	67
	[33]	179	48	16	76	104
	Total	409	47	17	154	255
Sweden	[19] ^a^	1000	51	17	442	558
Germany	[34]	2490	49	18	1204	1320
	[35] ^b^	578	39	12	90	488
	Unpublished data of German general population; follow-up study of [36]	199	48	17	121	94
	Total	3267	47	-	1389	1878
England	[12]	350	32	13	116	224
	[37]	150	29	11	80	70
	Total	500	31	13	196	294
Grand	Total	5176	46	-	2212	3024

*Note*. Age for the [34] subsample could not be obtained at the individual level. As such, the standard deviation for age in the German sample could not be computed (the presented mean is the weighted mean across samples).

^a^ Random subsample of original sample

^b^ Original sample containing: *n* = 474; extended sample refers to an online data collection between 10/2005 and 06/2008).

### Measures

The MHWS assesses concerns of respondents about modern environmental issues on a Five-Point Likert scale ranging from 1 (“no concern”) to 5 (“extreme concern”). All studies except for the Rief et al. [34] study used the original 25-item version. Rief and colleagues removed or changed the *Toxic interventions* items “fluoridation of water”, “vaccination programs”, and “bacteria in air condition systems”, as well as the Tainted food item “Pesticides in food”. Thus, we performed our analysis on the 21 common items across all samples (*Toxic interventions*: 8 items, *Environmental pollution*: 6 items, *Tainted food*: 4 items, and *Radiation*: 3 items).

### Statistical analysis

We will first outline which measurement models of MHWs and which measurement invariance levels we specified. We will then describe how absolute and relative model fit are evaluated before presenting the procedure for identifying the most suitable indicators.

#### Model specification

We modeled the MHWS as a higher-order factor model with a second-order factor (i.e. General MHWs) loading on four first-order factors (e.g. *Environmental pollution*), which in turn load on the items. We compared this model to the correlated factor model of MHWs, which is typically reported in the literature. We also compared the two models to a bi-factor model of MHWs, with the general MHWs factor loading on all items, and four (uncorrelated) nested factors (e.g. *Environmental pollution*) loading on the corresponding items of the scale. We also tested the latter two models with ESEM [24–26], which allows for cross-loadings in the model. Higher-order models are currently not implemented in ESEM, which is why we only tested this model in CFA. All models were estimated with the Weighted Least Squares Means and Variance adjusted estimator (WLSMV), which is suited for non-normally distributed categorical data. We used Theta parameterization, which provides parameters similar to SEMs with continuous data (e.g. residual variances).

#### Measurement invariance

We specified levels of invariance for the model by applying increasingly strict measurement parameter equality constraints across groups [38]. In the case of the higher-order model, constraints are first applied to the first-order and subsequently to the second-order factor level. In contrast to measurement invariance testing with continuous variables, the level of metric measurement invariance (i.e. equal factor loadings across groups) cannot be tested using categorical variables [39]. The item characteristic curves estimated for categorical measures are based on both factor loadings and thresholds, and have to be constrained simultaneously. The measurement invariance levels that can be tested in higher-order models are listed in Table 2. Note that scalar measurement invariance (i.e. equal factor loadings and thresholds across groups) or higher is required for the comparison of factor means across groups. Factor means were effect coded for identification (i.e. the sum of factor scores across countries was set to zero). Even though group sizes were imbalanced (see Table 1), which can increase Type I error rates of identifying violations of measurement invariance [40], we decided against reducing the sample size for the German sample, as this comes at the cost of reduced measurement precision and power [40], which we deemed more important in the current context.

**Table 2:** Measurement invariance levels for higher-order models with categorical data.

		1^st^-order factor level 2^nd^-order factor level						
First-order factor invariance level	Second-order factor invariance level	1^st^-order factor loadings	Item thresholds	Item residual variances	1^st^-order factor means (intercepts)	2^st^-order factor loadings	1^st^-order factor residual varinces	2^nd^-order factor means
Configural	Configural	*	*	1	0	*	*	0
Scalar	Configural	(Fixed	Fixed)	1/*	0/*	*	*	0
Strict	Configural	(Fixed	Fixed)	1	0/*	*	*	0
Strict	Metric	(Fixed	Fixed)	1	0/*	Fixed	*	0
Strict	Scalar	(Fixed	Fixed)	1	0	Fixed	*	0/*
Strict	Strict	(Fixed	Fixed)	1	0	Fixed	Fixed	0/*

*Note*. The asterisk (*) indicates that the parameter is freely estimated. Numbers indicate the value parameters are constrained to; Fixed = the parameter is constrained to equality across groups; Slash (/*) indicates that the parameter is constrained in one group (or on average across groups) for identification purposes and estimated freely in the other groups. Parameters in parentheses need to be varied in tandem (for additional remarks see text).

#### Model evaluation

We evaluated absolute model fit with the *Comparative Fit Index* (CFI) and the *Root Mean Square Error of Approximation* (RMSEA) based on common standards [41]. Invariance was tested using ΔCFI [42] between consecutive measurement invariance levels. The level of measurement invariance achieved was determined by absolute fit (CFI > .90 and RMSEA < .08) [41] and relative fit compared with the preceding step of invariance testing (ΔCFI < .01). We decided against using χ^2^-values to evaluate the models as the χ^2^-statistic is sensitive to sample size and non-normal distribution of the data [43]. Models were estimated in *Mplus* 7 [39].

#### ACO Item selection

We applied ACO in R [44] to find the indicators that would provide a measurement invariant comparison of the MHWS factors across countries. As such, we used ACO to optimize the CFI and RMSEA of the scalar first-order factor level, as well as the ΔCFI between configural and scalar measurement invariance at the first-order factor level. All three criteria were logit-transformed in order to (a) scale the range of the values between 0 and 1 and (b) maximize the differentiation around the critical cutoff values [29]. This also ensures that the three criteria are weighted equally. Based on the already high CFI values but problematic RMSEA, we transformed the values to maximize differentiation for the CFI around .98 and RMSEA around .07 (see Eqs 1 and 2).

$$\varphi_{CFI}=\frac{1}{1+e^{98-100CFI}}$$ (1)

$$\varphi_{RMSEA}=1-\frac{1}{1+e^{7-100RMSEA}}$$ (2)

ΔCFI was transformed with a cutoff at ΔCFI = .01, with lower values indicating an acceptable increase in model misfit between invariance levels (see Eq 3).

$$\varphi_{\Delta CFI}=1-\frac{1}{1+e^{1-100\Delta CFI}}$$ (3)

The overall optimization function was the sum of the transformed criteria (see Eq 4):

$$\varphi_{overall}=\varphi_{CFI}+\varphi_{RMSEA}+\varphi_{\Delta CFI}$$ (4)

For the short-scale, we selected the three indicators per factor that would maximize the given optimization function. Three was chosen because this number of manifest variables is the minimum for identifying the first-order factors. Retaining at least five items per construct has been recommended in order to maintain construct coverage [45]. However, this recommendation applies to broader and more overarching constructs. In the present context we select three items per sub-factor of MHWs, thus retaining twelve items to capture the entire construct. By maintaining the factor structure, we also ensure that construct coverage is minimally affected by item selection. For the ultra-short scale, we wanted to create the shortest possible measure of General MHWs that would still cover all four sub-factors. We thus select four indicators from the short-scale, with one indicator from each original factor. As the number of possible four item combinations based on the previously model was relatively small (81), we estimated all possible models and compared them based on the optimization criterion (see Eq 4). Selected items for both short forms and corresponding item characteristics are presented in Table 3. Translated short-scales for each country can be found in the online repository at https://osf.io/r9x6e/. The short forms correlated almost perfectly with the full 21-item version of the MHWS (short scale: r = .99; ultra-short scale: r = .96), indicating that construct coverage was maintained.

**Table 3:** Selected items and item characteristics.

Factor	Item	*M*	*SD*	Skew.	Kurt.	Std. Loading
Toxic Interventions	2	2.58	1.45	0.37	-1.26	.80; .83; .85; .73
	6*	2.65	1.30	0.29	-1.06	.86; .88; .90; .81 (.81; .84; 86; .86)
	9	2.70	1.38	0.24	-1.19	.82; .85; .87; .77
Environmental Pollution	14	2.95	1.27	0.03	-1.06	.83; .85; .86; .79
	15	2.82	1.21	0.07	-0.95	.90; .91; .92; .87
	16*	2.80	1.19	0.13	-0.89	.92; .93; .94; .89 (.75; .78; .81; .68)
Tainted Food	19	2.84	1.25	0.12	-0.99	.83; .86; .88; .79
	21	2.97	1.37	-0.01	-1.24	.96; .97; .97; .95
	22*	2.95	1.38	0.01	-1.25	.96; .97; .97; .94 (.83; .85; .88; .78)
Radiation	23	1.92	1.00	1.04	0.62	.84; .85; .87; .81
	24*	1.97	1.06	0.99	0.29	.95; .96; .96; .94 (.63; .67; .71; .56)
	25	2.06	1.12	0.88	-0.07	.88; .89; .90; .86

*Note*. *M* = Mean; *SD* = Standard Deviation; Skew = Skewness; Kurt. = Kurtosis; Item scores range from 1 to 5. Std. Loading = Standardized loadings of the short scale in the Hungary, Germany, Sweden, and England group under strict measurement invariance. Standardized loadings of the ultra-short scale under strict measurement invariance for the corresponding groups are printed below in parenthesis. Numbers in the item column represent the item number in the original scale. Items from the ultra-short scale are marked with an asterisk (*). Items for the scales can be found in the online repository at https://osf.io/r9x6e/. “Other environmental pollutions” needs to be relabeled as “Environmental pollution” in the ultra-short scale.

## Results

We first tested the different factor models on the full 21-item scale under configural measurement invariance across the four countries (see online repository at https://osf.io/r9x6e/ for fit indices and full loading structure of all models). The correlated factor model fitted the data worst (CFI = .974; RMSEA = .106). The correlations between the factors were similarly high as in previous studies (r = .55 - .89; on average r = .73). Even though the higher-order model is more parsimonious, it fitted the data slightly better, but still yielded insufficient model fit (CFI = .975; RMSEA = .103). The second-order factor loadings were on average .87, which supports the presence of a second-order factor. The bi-factor model of MHWs resulted in better fit (CFI = .984; RMSEA = .087), but suffered from a large number of negative or low loadings on the *Toxic Intervention* factor and a lack of robustness across countries. Given the large proportion of low factor loadings and difficult interpretability of the factors, we decided against using this model [46]. This problem was also apparent in the bi-factor ESEM model, which otherwise fitted the data well (CFI = .994; RMSEA = .064). However, as noted before, the scale scores can only be meaningfully interpreted when model fit, factor loadings and the theoretical foundation of the model are sound. In the case of the bi-factor model, the interpretability of the factors and corresponding scale scores was problematic due to the unclear factor pattern. While the ESEM correlated factor model yielded better model fit (CFI = .989; RMSEA = .081) than the CFA counterpart, a large number of cross-loadings were significant (151 out of a total of 252). Only 16 out of 63 possible cross-loadings per country were robust across countries (i.e., always or never significant). Note that despite being reduced by the inclusion of cross-loadings, the factor correlations were still reasonably high (average r = .61). Despite the better model fit of the ESEM and bi-factor models, we decided retain the theoretical model of MHWs and remove problematic items (instead of adding additional parameters to reduce misfit). We thus applied the higher-order factor model, which was the most parsimonious model and yielded the strongest factor loadings. However, the unsatisfactory RMSEA levels under configural measurement invariance indicated that the full scale does not adequately represent the theoretical structure of MHWs. As such, higher levels of measurement invariance across countries were not tested. Using ACO, we improved the absolute model fit of both short scales beyond the fit of the original scale (see Table 4). To account for possible effects of item reduction on the fit indices (i.e. improving model fit due to reduced model complexity), we compared the ACO models to item selection by chance. We randomly selected 1,000 12-item models and computed the 1^st^ and 99^th^ percentile of CFI, RMSEA and ΔCFI to examine the distribution of model fit (configural measurement invariance: CFI = .975-.994; RMSEA = .078-.132; scalar measurement invariance: CFI = .969-.990; RMSEA = .080-.117). As can be seen, ACO optimized absolute model fit for both measurement invariance levels beyond the 99^th^ CFI or 1^st^ RMSEA percentile of the model fit distribution.

**Table 4:** Measurement invariance testing of the short scales.

First-order factors	Second-order factors	Short scale			Ultra-short scale		
		*df*	CFI	RMSEA	*df*	CFI	RMSEA
Configural	Configural	200	.995	.076 [.072,.079]	8	.998	.076 [.060,.093]
Scalar	Configural	320	.992	.077 [.075,.080]	50	.989	.070 [.063,.076]
Strict	Configural	356	.991	.077 [.075,.080]	62	.984	.074 [.068,.080]
Strict	Metric	365	.992	.072 [.069,.074]	-	-	-
Strict	Scalar	374	.990	.079 [.077,.082]	-	-	-
Strict	Strict	386	.990	.078 [.075,.080]	-	-	-

*Note*. CFI = Comparative Fit Index; RMSEA = Root Mean Square Error of Approximation; 90% Confidence Interval of the RMSEA is given in brackets next to the RMSEA value. The highest measurement invariance level reached is underlined. The short scale contains twelve items measuring four first-order factors loading on a common second-order factor. The ultra-short scale contains four items measuring one factor.

**Fig 1:**
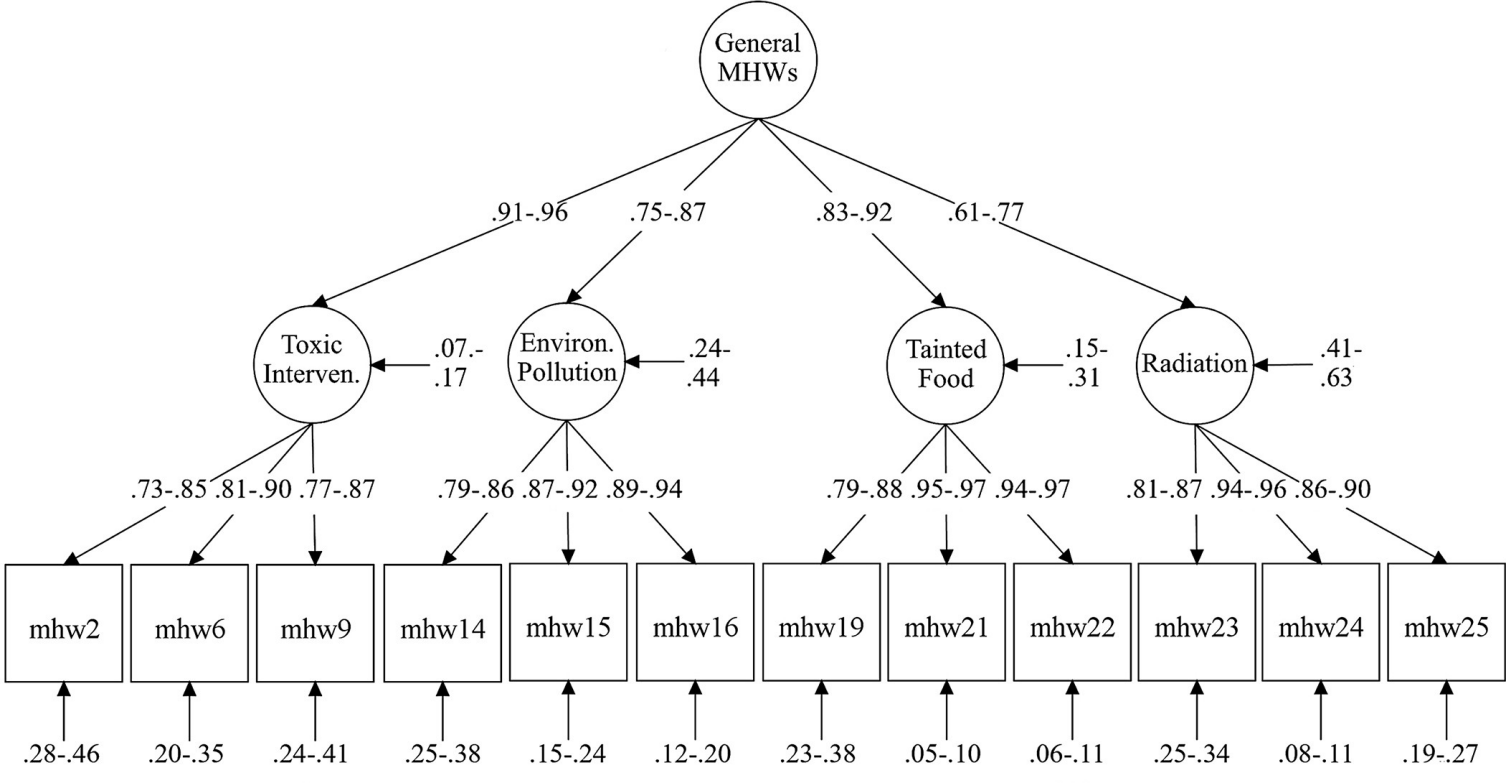
Higher-order model of the short scale. Range of standardized parameters across countries under strict measurement invariance (CFI = .990; RMSEA = .078; see Table 3 for full list of loadings).

**Fig 2:**
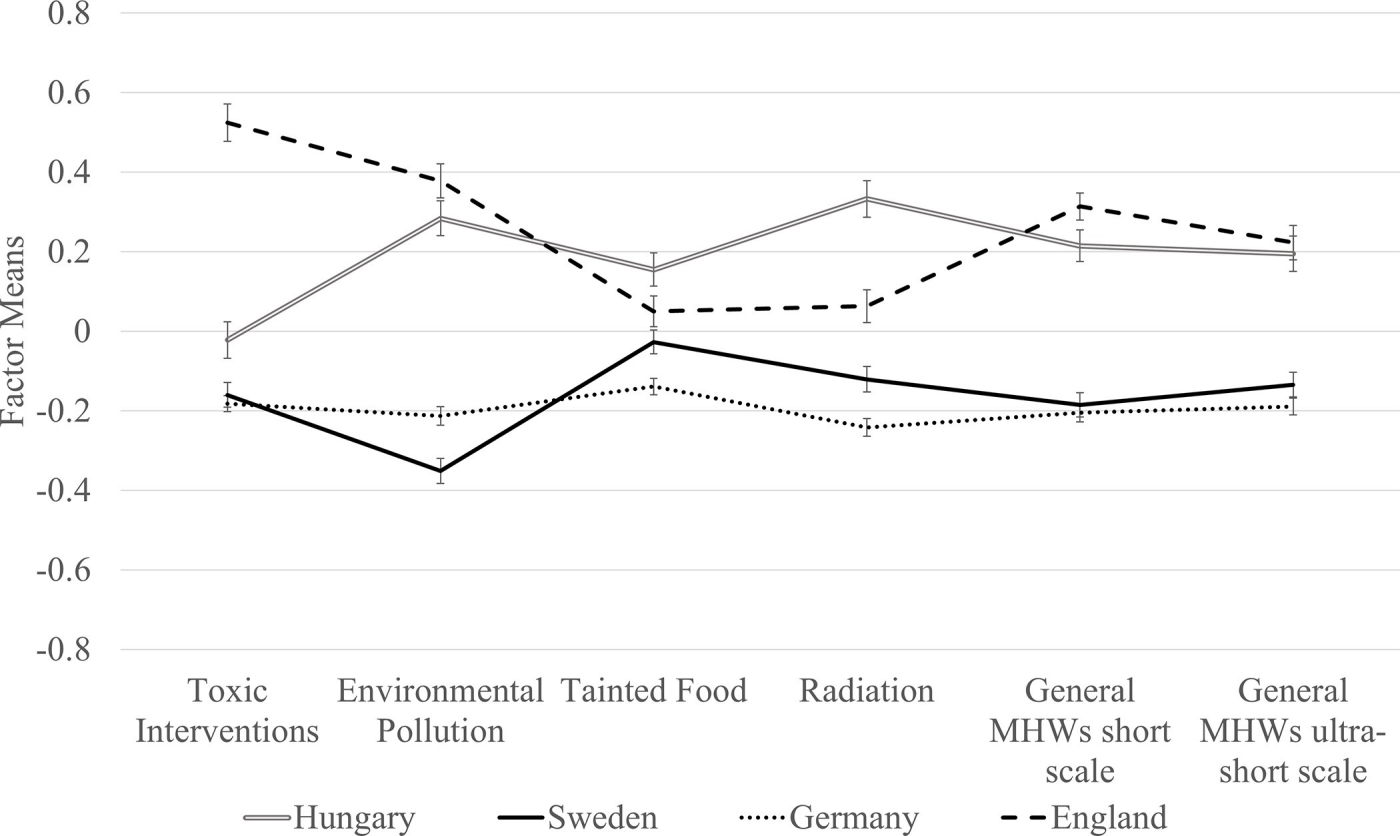
Factor means of the short scale across countries. The factor means are standardized z-values. Factor means were effect coded for identification (i.e. sum of factor means across countries is zero). Error bars represent one standard error in both directions. *Toxic interventions*, *Environmental pollution*, *Tainted food* and *Radiation* means were derived based on the model with strict measurement invariance at the first-order factor level and configural measurement invariance at the second-order factor level. The general MHWs mean of the short scale was derived based on the 12-item model with strict measurement invariance at the first-order factor level and strict measurement invariance at the second-order factor level. The general MHWs mean of the ultra-short scale was derived based on the 4-item model with strict measurement invariance.

The acceptable overall model fit and low ΔCFI levels of both short-scales supported the strict level of measurement invariance. Equal factor loadings, item thresholds and residuals across countries allow for an unbiased comparison of the factor means across countries. The selected model for the short scale under strict measurement invariance is presented in Fig 1. Factor means of the two short scales across countries are presented in Fig 2. The factor means of the overall MHWs factor did not differ between short forms. As strict measurement invariance was achieved by both short-scales, manifest scale scores could also be compared across countries (see Table 3).

Factor loadings were medium to high in both short forms (for details, see Fig 1 and Table 3) and resulted in adequate factor saturation McDonald’s *ω* across countries (short scale: *ω*_*ToxicInterventions*_ = .86-.91; *ω*_*EnvironmentalPollution*_ = .91-.93; *ω*_*TaintedFood*_ = .94-.96; *ω*_*Radiation*_ = .92-.93; *ω*_*MHWs*_ = .90-.93; ultra-short scale: *ω*_*MHWs*_ = .84-.89; *ω*s were computed based on the strict measurement invariance level).

Residents from all four countries show MHWs to at least some degree (for manifest scale scores across countries see online repository at https://osf.io/r9x6e/). Germany and Sweden yielded the lowest overall values of MHWs. Differences between these two countries were small across all factors. The Hungarian and English sample yielded the highest overall scores on the general MHWs factor. While the level of modern health worries was similarly high across the two countries, the main reason for concern differed substantially between these two countries. The English sample exhibited much larger concerns about *Toxic interventions* than samples from the other countries. Hungarians were more concerned about *Radiation* than participants from the other countries. Participants from both these countries show higher worries about *Environmental pollution* than participants from Germany and Sweden.

## Discussion

ACO was successful at improving model fit and measurement invariance of the modern health worries scale (MHWS) on a cross-cultural sample, while also maintaining the factor structure of the measurement. We developed two short forms that provide a valid and measurement invariant measurement of the construct across four European countries, namely Hungary, Sweden, Germany and England. We then compared the levels of the various facets and the general factor of MHWs across these countries.

The short version of the MHWS represents the four-factor structure of the original version very well with adequate model fit, decent factor saturation, and the highest level of measurement invariance across countries. Removing non-invariant indicators in order to establish measurement invariance has been criticized for narrowing the construct coverage [42]. The correlation between the short scales and the 21-item version was very high (short: r = .99; ultra-short: .96), indicating that construct coverage was not affected by the item reduction. However, the full scale did not yield satisfactory model fit and could hence not be used for a meaningful comparison of MHWs levels across countries. Given the high correlation between short and long form, as well as the differences in model fit, ACO only removed problematic and redundant items. Arguably, maintaining the four-factor structure resulted in the high correlation between the scales. This also applies to the ultra-short (4-item) scale, where we kept one item from each factor. Alternatively, the correlation between long and short form can also be optimized using ACO to ensure that construct coverage is maintained [47]. However, we advise caution when doing so: The correlation between long and short scale is only worth maximizing when the full scale represents a gold standard of measuring the construct (i.e., fulfilling all relevant psychometric criteria, such as content validity of the item sample, model fit, and reliability). We recommend using an approach similar to the one applied in this study (i.e., maintaining construct coverage by retaining the somewhat supported factor structure).

Both short MHWS versions indicated considerable differences between countries with respect to total scores and sub-scales. Please note that the short form should be preferred over the ultra-short form when feasible, as the ultra-short form only allows capturing differences at the general MHWs factor level across countries.

Worry about harmful consequences of electromagnetic radiation (e.g. mobile phones) was the highest in Hungary, while possible negative effects of toxic interventions (e.g. dangerous chemicals in household products) evoked excessive concern in England. Moreover, overall concern (total MHWs) as well as worry about various forms of environmental pollution (e.g. depletion of the ozone layer) were lowest in Germany and Sweden.

These findings are surprising and counter-intuitive, as both Germany and Sweden are famed for their environmental consciousness. Apparently, the primary motive behind environmentally conscious thinking is not health related worry but fears of further degenerations of the biosphere. It is also important to keep in mind that the Hungarian and British samples are not representative of the respective populations, while the German and Swedish data-sets are close to representativeness. Therefore, self-selection effects (in terms of an overrepresentation of participants with higher MHWs) might have been stronger in the English and Hungarian sample. Also, subtle differences in the framings of the study aims might have contributed to the national differences.

Another meaningful difference is that a marked political drift to the right took place in Hungary and England in the last decade, and the connection between political extremism (at both the left and the right) and conspiracy theories is well-known [48]. As MHWs are also connected to conspiracy theories and the intuitive-experiential thinking style that provides the basis for such beliefs [13], the political milieu can at least partly explain the high levels of general MHWs. This explanation might also be valid for the high *Radiation* scores, as physical characteristics of electromagnetic radiation (e.g. it is insensible for humans and can permeate the body) are particularly threatening for laypeople and might trigger intuitive-experiential thinking style [13].

The high level of worry about environmental pollution in Hungary and England may possibly be explained by specific health conditions. In a recent survey [49] citizens were asked how serious they considered air quality related health problems to be in their country. Both respiratory and cardiovascular diseases were rated by Hungarians and British as more serious than by Germans and Swedes. Because experimental studies demonstrated the ability of news reports to directly affect individual levels of MHWs [50], it is possible that national differences in coverage of environmental illnesses contributed to observed differences between countries.

We used ACO in this study to optimize the MHWs scale, as even model fit at the configural level was insufficient to compare factor scores across countries. The Alignment [51] technique has been proposed as an alternative to the overly strict MGCFA measurement invariance testing when measurement invariance is violated. Instead of constraining all indicators to equality across age, Alignment tries to minimize non-invariance on the majority of indicators by maximizing it on a specific subset of items (similar to exploratory factor analysis rotation techniques). By doing so, model fit at higher measurement invariance levels still corresponds to the configural level. Another alternative procedure to the one presented here is item selection based on *differential item functioning* (DIF). We chose ACO in this study, because neither Alignment nor DIF item selection will address issues of absolute model fit in the configural model, which was the main reason we shortened the scale. While item selection based on modification indices can also be used to improve model fit or measurement invariance–by identifying the item with the highest sum of modification indices, removing it, and re-estimating the model–this procedure and DIF selection rely on item-level characteristics to optimize scale score criteria, suffer from sequence effects, and are difficult to combine with other criteria (e.g., factor loadings, reliability). ACO can balance several scale and item level criteria simultaneously and uses a simultaneous item drawing procedure to eliminate potential sequence effects, which is why we suggest it as a solution when the full scale does not adhere to the psychometric requirements of the study.

ACO is a very flexible selection heuristic can be applied to any type of model (e.g., correlated, bi-factor, higher-order) and modeling framework (e.g., CFA, MGCFA, ESEM, IRT). Note that these decisions will strongly affect the final solution, as ACO will try to identify the best items within the given framework and optimization function. In an ESEM context, ACO will be less likely to remove items with cross-loadings than in the stricter CFA setting. Model misspecifications will also have a severe impact on the final solution: If a one-factor model is specified even though the true underlying structure consists of several correlated factors, ACO will select items that support the one-factor solution, thus narrowing the construct. As such, the framework and model used should be well considered before applying ACO or any other item selection procedure. This also applies to the optimization criterion. Typically, ACO will be applied when the full model does not meet the required psychometric criteria associated with the modeling framework. Which fit indices and cut-off criteria to use is still a matter of debate as many commonly used fit indices (e.g., CFI, RMSEA) have shown to be susceptible to the magnitude of factor loadings [46,52–54], the type of model specified [55,56] and the estimator used [57]. The decision on which fit criteria to use is thus always associated with the preferences of the researcher. This critique also applies to our decision to maximize CFI and minimize RMSEA and ΔCFI between measurement invariance levels. In our study, CFI and RMSEA values of all estimated models (including the random selection) were relatively high, indicating a discrepancy between the two model fit indices. Even though both fit indices rely on the χ^2^-statistic in some form, the relatively high factor loadings found in our study (on average λ = .83 for the full higher-order model; average λ = .89 for the 12-item short model), might have affected both indices differently. While the CFI may improve with higher zero-order correlations and subsequently worse null model [52], the RMSEA seems to show a different trend, also known as the *reliability paradox* [53,54]. When only optimizing model fit–thus not having to balance several criteria–a reasonable alternative to using a broad set of fit indices might be minimizing the χ^2^-statistic instead.

A limitation of this study was the restriction to 21 items instead of the original 25 item scale [34]. However, three of these items were removed or modified from the *Toxic interventions* factor, which was still overrepresented with a total of eight items in comparison to only three to six indicators on the other factors. Hence, we believe that the impact of the reduction of *Toxic interventions* items does not affect the subsequent analysis in a substantial way. The only item that was removed from another factor, “pesticides in food” from the *Tainted food* factor, was removed by Rief and colleagues [34] due to its similarity to the Environmental pollution item “pesticide spray”. Concerns about pesticides were hence still covered in our 21-item pool. Another limitation of the findings is the use of varying data collection procedures (i.e. paper-and-pencil and online methods) in the different national studies.

## Conclusion

ACO has shown to be an adequate tool for improving measurement invariance in a cross-cultural setting. The short (12-item) version of the MHWS maintains the original four-factor structure of the original scale, while also yielding good model fit and measurement invariance across the four countries assessed in this study. The ultra-short (4-item) scale is appropriate for the measurement of the general construct, but is unable to detect meaningful differences across countries at the factor level. In addition, the reduced item number will negatively affect measurement precision. We hence recommend maintaining the original factor structure when shortening measurement inventories. This can be done easily using ACO, as it will select item sets of a fixed size for a pre-defined model instead of removing items sequentially. In this study, the English and Hungarian sample showed higher levels of MHWs than the German and Swedish sample. While general levels of MHWs were similar between the Hungarian and English sample, Hungarians seemed to be more concerned about *Radiation* than the other countries. Participants from England were more worried about *Toxic interventions*. Concerns about *Environmental pollution* were high in both these countries. General levels of MHWs were similarly low for German and Swedish participants.
